# Food Impaction in Dentistry: Revisited

**DOI:** 10.3290/j.ohpd.b4172837

**Published:** 2023-06-22

**Authors:** Van Mai Truong, Soyeon Kim, Yang-Jin Yi, Young-Seok Park

**Affiliations:** a Postgraduate Student, Department of Oral Anatomy and Dental Research Institute, School of Dentistry, Seoul National University, Seoul, Republic of Korea. Literature search, wrote the manuscript.; b Postgraduate Student, Department of Oral Anatomy and Dental Research Institute, School of Dentistry, Seoul National University, Seoul, Republic of Korea. Literature search.; c Professor, Department of Prosthodontics, Section of Dentistry, Seoul National University Bundang Hospital, Seongnam, Republic of Korea; Department of Dentistry & Dental Research Institute, School of Dentistry, Seoul National University, Seoul, Korea. Provided clinical photos, reviewed the manuscript.; d Professor, Department of Oral Anatomy and Dental Research Institute, School of Dentistry, Seoul National University, Seoul, Republic of Korea; Head of Center for Future Dentistry, School of Dentistry, Seoul National University, Seoul, Republic of Korea. Wrote the manuscript, supervised the study.

**Keywords:** food impaction, periodontitis, proximal space, proximal contact, restoration

## Abstract

**Purpose:**

This review aimed to highlight the aetiology and risk factors of food impaction along with the treatment in each case.

**Materials and Methods:**

A search was conducted in PubMed from 1947 to March 28, 2023. The search terms utilised included (food impaction) OR (interdental impaction). No filter was applied. Articles related to the classification, aetiology, treatment, and associated factors of food impaction in dentistry and published in English or with an abstract in English were selected.

**Results:**

A total of 72 articles were included in the review, which revealed the variety and complexity of aetiological factors and treatment of food impaction in dentistry, as well as the heterogeneity of previous studies. Based on the aetiology, different treatment plans and management should be considered.

**Conclusion:**

This review indicated the need to identify the pathology of food impaction before treatment. Considering the causal factors of food impaction – including proximal contact loss, occlusal disharmony, morphological deformity, positional abnormality, and interdental papillae loss – different management approaches such as restoration, occlusal adjustment, orthodontic, nonsurgical or surgical treatment could be applied. Further clinical and experimental research is warranted to address the prevention and treatment of food impaction in dentistry.

Food impaction in dentistry is described as the forceful wedging of food debris into the proximal space by occlusal, lip, tongue, and/or cheek pressures during mastication.^45,54,126^ Open tooth contact, marginal ridge integrity, plunger cusp mechanisms, occlusal wear, extrusion beyond the occlusal plane, congenital morphological abnormalities, periodontal recession, etc, are considered aetiological factors of food impaction.^20,41,46,64,96^ The impacted food might irritate the adjacent tissue and lead to various oral problems such as proximal caries, gingivitis, periodontitis, and periodontal atrophy.^20^ Moreover, this impaction is related to oral malodour, discomfort, and pain, which substantially affect patients’ daily life.^8,41,94,102,109^ Thus, the treatment, management, and prevention of food impaction in dentistry are necessary. However, owing to the variety of aetiological factors, the cause of food impaction should be addressed first, and a specific treatment plan should be established afterward based on the cause.

In the literature, several studies have attempted to evaluate the aetiology, related factors, or treatment of food impaction in an individual setting. However, these studies are generally from anecdotal points of view, involve unstandardised experiments, or present statements based on personal clinical experience. In addition, the pathology and management of this condition have not been comprehensively explored. Thus, this review aimed to summarise and synthesise the aetiology and potential factors favouring food impaction in dentistry and simultaneously suggest treatment in each situation based on published evidence.

## Materials and Methods

The PubMed database was searched for this review. The following search terms were utilised for data search: (food impaction) OR (interdental impaction). The search of the database covered the timespan from 1947 until March 28, 2023. No filter was applied. The title, abstract, and full text of returned records were manually and individually screened. Studies associated with the classification, aetiology, related factors, or treatment of food impaction in dentistry and published in English or abstract in English were included.

## Results

**Fig 1:**
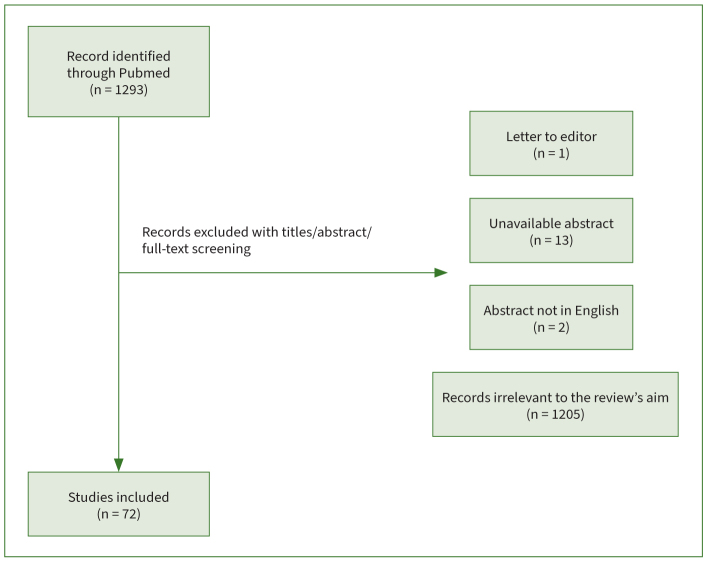
Identification of included articles.

The search results are illustrated in Fig 1; the literature search yielded a total of 1293 studies. After screening the title, abstract, and full text of the identified studies, excluding letters to the editor, articles without abstracts or with non-English abstracts, or articles irrelevant to the review’s aim, a total of 72 articles were selected for this review. These demonstrated the variety and complexity of the aetiology of food impaction in dentistry. The aetiolgical factors can be classified into proximal contact loss (PCL), occlusal disharmony, morphological deformity, positional abnormality, and periodontal recession. In addition, related factors might contribute to the severity of food impaction. Thus, depending on the causative factors, different treatment is suggested. However, the heterogeneity and subjectivity of the studies make it difficult to standardise a treatment plan.

### Classification

**Fig 2:**
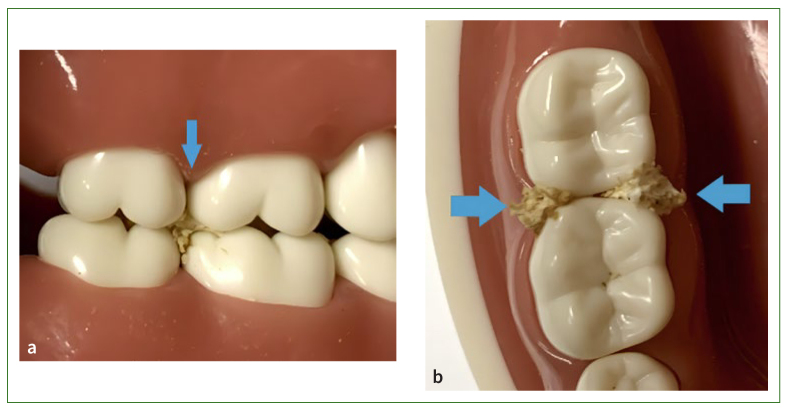
Vertical and horizontal food impactions. (a) Vertical food impaction. (b) Horizontal food impaction.

Food impaction could be generally divided into vertical or horizontal impaction, based on the direction of the impaction (Fig 2).^45,54^ However, it may also occur simultaneously in both directions.^96^ Vertical impaction is caused by occlusal pressure wedging the food debris into the proximal space, whereas horizontal food impaction results from lateral force from the cheeks, tongue, and lips pressing food plaque into the periodontal recession.^64^ Vertical food impaction was reported to cause acute gingivitis or gingival abscess more regularly, be more destructive to the periodontal tissue and more uncomfortable for patients, but less complicated to treat than horizontal food impaction.^46,69^

### Pathology

Proximal embrasure is defined as a V-shaped space created by the curved proximal profiles of two adjacent teeth in the same arch. Four proximal embrasures surround the proximal contacts, i.e. gingival, occlusal (incisal), buccal (labial), and lingual embrasures. Among them, occlusal, buccal, and lingual embrasures serve as spillways for food debris to escape during mastication. In the optimal situation, the interdental papilla completely occupies the space between teeth created by the gingival embrasure.^48^

**Fig 3:**
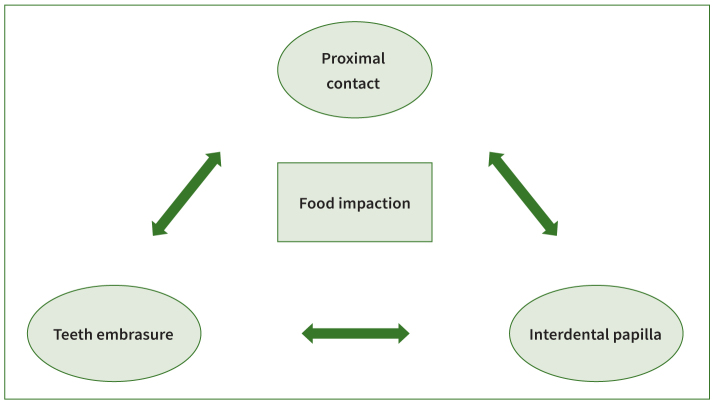
Pathology of food impaction.

Altogether, the relationship between proximal contact, teeth embrasure, and interdental papilla is the main factor in preventing food impaction. In other words, any deformity, abnormality, or malposition in interproximal contact, embrasures, and interdental papilla will likely lead to food impaction (Fig 3).

### Aetiology

#### Vertical impaction

The continuity of the teeth in the dental arch, appropriate contact area, and existence of grooves and marginal ridges are considered the natural protective mechanism to avoid vertical food impaction.^68^ Vertical food impaction has a multifactorial aetiology, which can be divided into proximal contact loss (PCL), occlusal disharmony, morphological deformity, and positional abnormality.

##### Proximal contact loss

PCL is the most common factor associated with vertical food impaction.^96^ An optimal proximal contact is essential to avoid not only food impaction and periodontal complications but also tooth migration.^41^

During mastication, a tooth is pressed downward into the alveolar bone and in contact with its adjacent teeth. After the functional process, they return to their initial position.^52,85^ During clenching, the teeth are dislocated and contact improperly with the neighbouring teeth. The repetition of this process can cause proximal wear between teeth, flatten the contact area, lead to open proximal contact formation, or create room for the teeth to move.^85,100^ The degree of this proximal wear and maintenance of tight proximal contacts were reported to be related to the frequency and extent of mesial drifting,^52^ because the teeth tend to move mesially during adulthood, driven by the anterior component vector provided by the occlusal force. Furthermore, high occlusal force was shown to possibly enhance mesial drift.^118^ Overall, the occlusal force, mesial migration, and PCL interact with each other. In addition, the tightness of proximal contact is also affected by other factors, such as musculature, periodontal ligament, and soft tissue force.^96^ Moreover, the posterior component force exists along with the anterior one. However, the posterior component is five times smaller than the anterior component.^110^ This might explain why the mesial proximal wear is faster than the distal proximal one, creating a mesial concavity.^49^ Furthermore, craniofacial growth during adulthood can alter the occlusal relationship and proximal contacts.^27^ These forces might be related to tooth movement over time.^119^

PCL can occur between implant-supported fixed dental prostheses (IFDP) and the adjacent teeth after restoration placement, even if the proximal contacts are restored properly. The most widely accepted explanation for this phenomenon is the mesial and distal drifting of the natural teeth, as mentioned above, that do not affect the position of the implant-supported prosthesis.^13,42,72,84,117^ Without periodontal tissue, implant prostheses more closely resemble an ankylotic tooth, do not migrate, and have much less vertical movement during function compared with natural teeth.^44,66,84^ Thus, the interproximal contact point is opened by the movement of the adjacent teeth away from the implant. Studies have shown that PCL can occur on mesial and/or distal aspects. However, mesial contact has a higher risk of PCL development.^1,60,111^ This higher susceptibility might be explained by the higher anterior component of force than the posterior component force. Although not all patients with PCL are aware of food impaction, approximately 40% reported having constant food impaction in these areas.^12,111^

Many factors can affect the PCL between the implant prosthesis and the adjacent natural teeth, e.g. patient-related factors (age, sex, and routine care), location-related factors (maxilla/mandible and premolar/molar), neighbouring tooth-related factors (occlusal force, endodontic treatment, splinted teeth, and single root/multiple roots), and bone-related factors (level of bone loss and bone quality).

Regarding patient-related factors, the rate of proximal contact loss was higher in older persons, which may be associated with the increase in tooth movement in this age group;^87,119^ however, this was not in agreement with a study by Bompolaki et al,^12^ who did not find statistically significant differences. Sex might also be a factor related to proximal contact, and women were found to have tighter mesial and distal proximal contacts than men.^12,35^ Routine dental care may also be a risk factor for PCL. Flossing might not be generally suggested to manage food plaque, except for the sites where the interdental space is too narrow for an interdental brush to access with no damage,^61^ because its technique is somewhat too demanding to be performed effectively. The interproximal brush is considered the most efficient way for controlling interproximal plaque.^7^ Nonetheless, the use of these brushes more than twice a day influences PCL; however, the use of a floss stick or dental floss did not statistically significantly affect mesial PCL.^66^ It might be caused by excessive force leading to greater tooth drifting than in individuals who do not use interdental cleaning aids or only do so once a day. Moreover, inappropriate proximal hygiene can cause traumatic lesions such as inflammation or ulcers, which may lead to more severe food impaction.

Concerning location-related factors, the prevalence of PCL was reported to be slightly higher for mandibular than maxillary prostheses;^12,35,60^ however, this result was not in agreement with the findings of Varthis et al.^111^ This higher incidence is believed to be related to root angulation and continuous mesial migration of the teeth, which is more pronounced in the mandible.^28^ IFDP inserted in premolar sites showed tighter mesial proximal contacts than those inserted in molar sites.^12^ These findings are possibly explained by the natural mesial drifting of adjacent teeth, which occurs most frequently in premolars but occasionally also in canines.^51^

In terms of neighbouring tooth-related factors, PCL was seen less in an IFDP occluding the removable partial dentures, which might be due to the softness of the material surface, as a lower occlusal force is less likely to change the proximal and occlusal contacts.^60^ An endodontically treated tooth next to an IFDP raised the PCL rate;^60^ however, this did not agree with the findings of Bompolaki et al.^12^ Koori et al^60^ found that when the adjacent teeth are splinted, the PCL at the mesial surface might decrease. In addition, the PCL incidence of the splinted IFDP was higher than that of the single IFDP.^13,60,66,119^ Furthermore, root configuration has been reported as another related factor. Adjacent teeth with a single root were found to be statistically significantly associated with a higher risk of PCL development.^87^

For bone-related factors, reduced bone support and bone quality were directly proportional to the incidence of PCL.^87,114^ Poor bone quality and support will be more affected by occlusal forces and might lead to tooth movement farther away from the IFDP.^114^

However, PCL did not appear in all regions in patients who received IFDPs in more than one region.^66^ There may be other related factors because of the complexity of the occlusal relationships and the masticatory system.

Other potential causes of PCL include inappropriate proximal contacts of teeth or restorations, habits of pushing teeth out of position, adjacent teeth drifting after extraction as a result of non-replacement of a missing tooth, and periodontal disease.^64^

##### Occlusal disharmony

**Fig 4:**
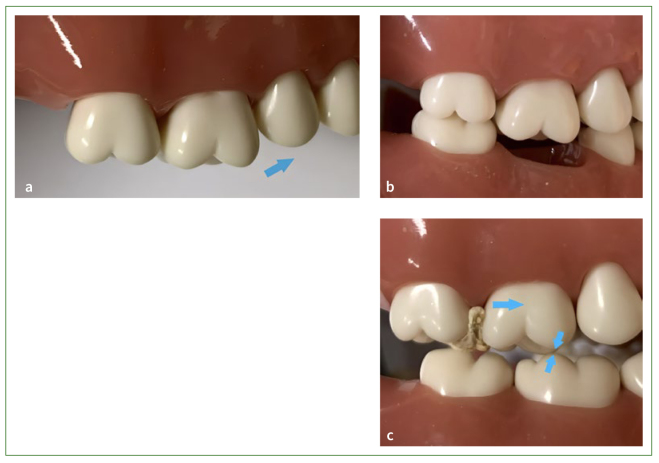
Occlusal factors of food impaction. (a) Uneven marginal ridge. (b) Extrusion beyond the occlusal plane. (c) Unstable contact due to occlusal interference.

Tight, well-positioned contacts do not always assure that teeth or restorations will be free of food impaction.^81^ This kind of food impaction is also known as kinetic food impaction, related to the occlusal factors including occlusal wear, lack of food escape grooves, uneven marginal ridges, extrusion beyond the occlusal plane, occlusal interferences, and unstable contacts (Fig 4).^25,64^

Undesirable guiding or balancing interferences can affect contact stability during mastication, and this may cause kinetic food impaction on stable posterior teeth. When the teeth are occluded, occlusal interference causes distal rotation of the posterior teeth, opening the proximal contact and allowing the food debris to penetrate the embrasure site. When the occlusion is released, the teeth move back to their original position, and food debris is trapped in the embrasure area.^120^

In addition, small marginal ridges and a lack of food escape grooves increase the possibility of food being forced into the embrasure site instead of the occlusal surface.^120^ Moreover, dentition wear will increase the occlusal contact area and create sharp, small plunger cusps. During chewing, these cusps are susceptible to food accumulation because of wedge-shaped extrusion.^20^ In addition, food impaction was reported to be statistically significantly related to bruxism and sharp tooth edges, leading to tooth decay and tooth pain.^37^

Furthermore, occlusal step deformity between the marginal ridges of teeth is formed when an uneven margin ridge and extrusion are beyond the occlusal site. This creates inappropriate proximal contacts between these teeth and provokes food impaction.^54^

##### Morphological deformity

A potential causative factor of food impaction is a defective tooth or restoration with any abnormal morphological characteristics in size or shape.

In restorative treatment, if the principles of proximal contact relationship, margin placement, and contour could not be followed successfully, food impaction occurs.^53,54,79,89^ Restorations with inappropriate contact areas, faulty facial and lingual contours, and overhanging or deficient margins often contribute to food impaction, making it more difficult for the patient to maintain good hygiene.^32,79^ Hence, this will affect the surrounding tissue, induce caries in the abutment and adjacent teeth, and periodontal inflammation, which can subsequently lead to prosthesis failure.

A proximal contact is faulty when the following features are present: flat, open, tight, rough surface, or poorly polished.^68^ Besides food impaction, inappropriate contact might result in tooth movement and rotation, disturb the axial relationship leading to trauma, disturb the coordination of the inclined cusps and planes causing deflective occlusal contacts, and injure tissues.^68^ Protecting the interdental papillae is one of the most important functions of proximal contacts; however, neither tight nor open contact can perform this function because of its conduciveness to food accumulation. Tight contacts make it more difficult for the patients to access the area for oral hygiene and are strongly associated with carious lesions. However, open contacts may lead to other problems, such as adjacent tooth drifting or tilting.^32^ In addition, an overcontoured crown on proximal surfaces may be a cause of tight contact. It decreases the embrasure space, causing stress and papillary irritation, leading to gingival inflammation and inhibiting effective oral hygiene.^32,98^

A question that arises here is what defines a ‘proper’ proximal contact. In the literature, various methods have been suggested to assess the proximal contact, including tactile assessment employing dental floss, shim stock, orthodontic dynamometer, metallic articulating film, and noncontact displacement system. Boice et al^11^ suggested that proximal contacts should be adjusted until a shim stock of 0.0005 inches can slightly pass through the contacts and 2 shim stocks with the same size will hold. Other studies have recommended that a satisfactory proximal contact was achieved when a strip with a thickness of 50 μm could be inserted with moderate resistance.^60,118^ Among these methods of evaluating the tightness of proximal contact, the tactile assessment method with dental ﬂoss was considered easy and simple, in addition to being the most clinically relevant and least elaborate conventional method.^55,93^ However, using either shim stock or dental floss may bring variable outcomes depending on the user. Furthermore, because of the instability of proximal contact strength during function and parafunction, it cannot be considered a static element which can be easily determined intraorally.^52^ In addition, the use of different techniques in evaluating the degree of proximal contacts could also yield outcomes that differ between studies.

Nagarsekar et al^79^ reported that the posterior mandibular region was the area most susceptible to food impaction, followed by the posterior maxillary area. These outcomes are similar to the findings of Linkow,^68^ in which mandibular fixed partial dentures collected more plaque than did their maxillary equivalents, especially in the molar region. This study also showed that the proximal area was the most common site involved in food impaction.^79^ The proximal region was determined to be the primary area exhibiting caries and periodontal disease.^103^ However, in restoration fabrication, the interdental restoration surfaces have been assessed as the most neglected sites.

##### Positional abnormality

Any positional abnormalities that disturb the proximal relationship, such as lingually or buccally rotated teeth, tilted teeth, and partially impacted teeth are prone to food impaction. Tooth positional abnormality can be congenital, a result of non-replacement of a missing tooth, or second-molar tilting after third-molar extraction. The partially impacted mandibular third molar was also reported to cause food impaction, which eventually led to paradental cysts.^21,26^

#### Horizontal food impaction

Horizontal food impaction is mainly induced by periodontal recession that creates an abnormal space under the proximal contact area.^36^ In addition, this recession may create aesthetic and phonetic problems.^106^ Interdental papillae are often lost for various reasons, such as alveolar atrophy, periodontal diseases, trauma, and aging.^14,123^ After extraction or implant insertion, there should be no delay in prosthetic rehabilitation, because the papilla tends to recede and form an embrasure.^22^ Therefore, prompt prosthetic rehabilitation is a decisive factor to preserve the existing papilla and maintain the new embrasure space.^22^

Horizontal food impaction is believed to be more complicated to alleviate than the vertical type.^31^ This type of impaction is said to be more bearable than vertical food impaction, because food remains or fibers can be removed easily using a toothpick or dental floss, providing momentary relief.^64,96^ However, frequent use of interdental aids might not only provoke the existing irritation but also make the patients frustrated.^96^

Certain anatomic characteristics have been considered determining factors that regulate the presence of healthy, stable papillae. The distance between the base of contact and the bone crest was one of the most crucial factors.^56,126^ As the distance decreased, the likelihood of interdental papillae being present increased.^59,107^ In addition, the influence of this distance on the interdental papillae increases with age.^58^ Moreover, this distance had a different effect on central and lateral papillae.^78^ A positive correlation was found between this distance and age, while a negative correlation was found between age and papilla height.^14^ A larger proximal contact was proven to increase the probability of an interdental papilla being complete.^23^ Gingival thickness, an important factor, affects not only the presence of interdental papillae but also the effectiveness of interdental papilla reconstruction.^126^ A thick biotype is more likely to be related to shorter and flatter interdental papillae. In addition, it has a greater blood supply, more keratinised support, and better resistance.^126^ In addition, root angulation and proximal root distance may play some roles in the presence of papillae.^59,101^ Better knowledge of these related variables will allow better interdental papilla management.^48^

#### Congenital oral anomalies

Both congenitally missing and supernumerary teeth (e.g. tuberculate supernumerary teeth and mesiodens) can lead to food impaction.^74,75,108^ However, food impaction is not the chief concern in these cases; rather, malocclusion, poor aesthetics, and cyst formation represent some of the more severe complications. In addition, other congenital soft tissue abnormalities should be identified. A lip frenulum with excessive attachment has been considered to lead to periodontal diseases associated with food impaction, restrict lip movement, difficulty in effective oral hygiene, and orthodontic problems; frenectomy is needed to release the lip frenum.^29^

Aside from local occlusal disharmony, malocclusion was reported to increase the frequency of food impaction.^80^ A correlation was also found between malocclusion and periodontal disease.^92^ Malocclusion can cause periodontal lesions, such as in periodontal pockets and the sinus tract, which are favourable sites for food debris to be impacted. A case report emphasised the possibility of bone destruction caused by this impaction, particularly in patients with poor oral hygiene.^80^

In congenital cases, with or without food impaction, diagnosis and treatment should be provided as soon as possible for better outcomes. The treatment can be complicated and involve different disciplines, including orthodontic, restorative, and surgical procedures.^9,75,108^

### Complications

Food impaction eventually leads to plaque accumulation. Moreover, food plaque or debris is one of the fundamental causative factors for caries and periodontitis. Hence, food impaction is one of the common causes of decay as well as gingival and periodontal diseases.^54^ The perception of ambiguous pressure and pain, foul taste, gum bleeding, and halitosis have been reported by patients as symptoms of food impaction.^5,54,79^ Subsequently, it has been reported to negatively affect the surrounding periodontal tissue condition and increase the risk of caries, tissue inflammation, trauma, pocket formation, and loss of interdental bone.^12,32,41,46,54,72,79,111,119^ However, existing periodontal pockets could trap food debris, which aggravates the severity of the periodontal lesion and forms a bony defect.^80^ Some patients did not notice the presence of food impaction until its complications manifested.

### Diagnosis

Two methods can be utilised to detect and evaluate food impaction, including patient self-report and clinical assessment. The detection of food impaction also depends on diet, oral hygiene, and patients’ perception.^15^ Moreover, not all patients with food impaction are aware of it, and the absence of food remnants at the time of clinical examination cannot deny the possibility of its existence. Thus, clinicians should employ both methods.

### Treatment and Prevention

Food impaction is important in the development of carious and periodontal lesions. Lesions must be treated. Scaling and root planing is considered the nonsurgical gold-standard for periodontal treatment.^63^ Moreover, if food impaction cannot be eliminated, successful treatment cannot be achieved. Therefore, clinicians must detect, evaluate, and manage the causative factors of food impaction.

#### Proximal Contact Loss

Given that various potential factors have been indicated as contributing to PCL, it might be difficult to avoid all of them. The treatment for PCL is to establish new optimal proximal contacts by a conservative method or new full-coverage restorations such as fillings, onlays, inlays, and crowns.^39,81,88,90^ A study introduced a mesiodistally adjustable crown, which was believed to enable the formation of new contacts without restoration replacement.^17^ However, all fixed prosthetic treatments cause injury to sound tooth tissue to some extent, affect the prosthesis, and frustrate dentists and patients. Other simple and atraumatic techniques have been developed to close the proximal space, such as using fiber-reinforced chemical-curing resin^40^ or flowable composite resin aided by cerclage wire under tension.^64^ Although the patient was satisfied and the effectiveness was immediate, the materials used had low filler content, making them susceptible to fracture and wear, which may limit the restoration’s longevity.^64^

The PCL between IFDP and its adjacent teeth is a relatively frequent complication, which has been described in many publications.^12,13,15,35,39,50,60,66,71,99,111,112,118,119^ In the literature, the incidence of this PCL ranges from 27% to 66.9%. However, the exact time that PCL first occurs has not yet been confirmed; the earliest PCL reported was 3 months after prosthetic insertion.^39^ The different monitoring lengths and heterogeneity among studies could have some effect on the overall PCL prevalence and accuracy of reported PCL onset. Thus, prospective studies with shorter intervals between appointments are necessary to assess these elements.

A statistically significant negative correlation was found between proximal contact tightness and the follow-up time after prosthetic placement: the longer the follow-up time, the looser the proximal contact.^12,13,60,72,111,118^ Owing to the high incidence of PCL, patients must be informed of this event as a possible implant complication (a consent form including this information is recommended), and whether PCL becomes evident at follow-up visits must be carefully monitored.^12,39,60,71^ The use of screw-retained prostheses or temporary adhesives for cement-retained prostheses has been recommended to modify the proximal contact, because the use of permanent cement may affect the stability of the implant and abutment during removal.^12,39,111,117^ A study suggested slightly adjusting both adjacent sites of neighbouring teeth before taking impressions for implant prostheses, so that they are rounded and flatter in profile, favouring optimal proximal contacts.^39^ However, in screw-retained restorations, a minor rotation might lead to the modification of the contact area, which may exert unnecessary forces on the neighbouring teeth.^111^ In addition, screw-retained prostheses are not indicated in all situations. Moreover, the prosthesis may debond during function because of the low strength of provisional cement,^17^ and the residual excess cement might negatively affect the peri-implant tissue.^111,113^ Moreover, correcting the proximal contact of ceramic restorations which have been in function intraorally for an extended duration is time-consuming and technically difficult; prosthesis removal or veneering ceramic re-application may be required in some cases.^111^ The extent of the modification required, restorative materials, laboratory skills, financial aspects, and time have all been considered to affect the decision to replace or repair the restoration.^39,72^

Nonetheless, a long-term study showed that the recurrence incidence of mesial PCL was high and related to the splinted-type design (>50%), and the recurrence time increased after each repair.^67^ The unpredictability of the PCL between the IFDP and natural teeth makes it complicated to control.

To prevent the teeth from moving, an occlusal device or an Essix retainer has been suggested as an effective solution.^3,111,112^ Nevertheless, several studies have reported ambiguous results. In a study by Bompolaki et al,^12^ no relationship was found between the prevention of PCL and the use of the occlusal device. Meanwhile, other studies have shown that the incidence of open contacts was reduced after wearing an occlusal retainer for 1 year.^3,50,125^ However, this device is only useful to avoid PCL that is caused by tooth movement. In addition, patients with implants should be recalled every 3–6 months for careful monitoring and early intervention.^88^

Future investigations should be performed to analyse the risk factors and management of PCL, owing to its high prevalence and complexity.

#### Occlusal Disharmony

For this causative factor, occlusal adjustment could be considered the basic treatment to avoid or distribute stress on teeth. This may include occlusal recurving, proximal surface recontouring, adjusting, grinding, and filling of the cusp, creating grooves and shallow fossa to create a food escape, adjusting the functional area, and eliminating marginal ridge discrepancy.^20,39,64^

Newell et al^81^ presented an occlusal adjustment method to correct occlusal abnormalities. In their study, the inadequacy of food escape grooves was considered the most common occlusal factor causing food impaction, followed by uneven marginal ridges and prominent occluding cusps.^81^ In this method, lingual and buccal escape grooves were created at the position where the marginal ridges contacted the cuspal ridges to allow lingual and buccal food deflection.^81^ If present, prominent opposing cusps and irregular marginal ridges were also corrected; this technique successfully eliminated food impaction.^81^ In another study, a sequential occlusal adjustment was conducted and showed efficacy by decreasing the mortar-and-pestle–like cusp, creating a food-escape groove and reducing the mesial surface of the distal cusp.^122^ This technique can be applied not only on teeth but also on restorations. However, when employing this method, clinicians be careful not to expose the dentin or perforate the restoration. Furthermore, contouring the marginal ridges, accentuating the triangular fossae, and deepening the distal and mesial pits were suggested to provide divergence, which will guide the food onto the occlusal surface rather than into the proximal areas, which might also help reduce food impaction.^120^ In addition, correcting the wide-centric contacts to point-centric contacts, removing unnecessary working guidance, and eliminating balancing interferences were recommended to maintain the proximal contact stability.^25^

These therapies have the advantages of requiring less tissue removal, greater operative simplicity, and good acceptance by patients.^20^ However, this treatment is irreversible, and predicting the prognosis and deciding the extent and area of selective grinding to quantify the occlusal adjustment is difficult.^65^ Further studies are required to address the needed extent of these alternations. Recently, Cheng et al^20^ introduced a new research method using deep learning to evaluate the features of food impaction given tight proximal contacts; this method may present a promising research direction for the clinical treatment of food impaction.

#### Morphological Deformity

In the presence of a defect, such as decay, fracture, cervical lesions, and exposed furcation areas, restorative therapy should be selected based on the extent of the defect to re-establish the normal morphology and prevent further complications.^10,20^

In small tooth defects, composite fillings can be placed. However, in more serious defects, ceramic restorations are a preferred method, as they offer better surface smoothness, marginal fit, and recovery of occlusal function than do composite fillings.^18^ Nevertheless, with either fillings or ceramic inserts, a good proximal relationship should be ensured.

Concerning defective restorations, redoing fixed partial dentures to restore the ideal contour and contact was considered the preferential treatment for food impaction management by nearly all dentists.^54,79^ In addition, to avoid food impaction, reinforcing and prescribing appropriate interdental aids after reconstructing the defective fixed partial denture have been suggested.^79^ A provisional prosthesis with good contour was believed to be a predictable way to achieve a biocompatible restoration.^38^

Regarding proximal contacts, the recommendation was to place the contact as far occlusally as possible and buccal to the central fossa (except between maxillary first and second molars) to create a large embrasure for optimum health of the papilla, especially lingually.^7,97^ Nonetheless, the large embrasure may lead to horizontal food impaction and phonetic and aesthetic problems. Although it has been said that with undercontoured and open embrasure space, horizontal food impaction barely occurred as long as the proximal contacts were managed properly,^68^ this may also depend on patients’ diet and masticatory habits. When trying in the prosthesis, if the contact is too tight, the contact area can be ground slightly after being checked carefully with an articulating paper and then polishing thoroughly. If an open contact is present, porcelain should be added in the laboratory or the prosthesis remade.^79^

With respect to the prosthesis contour and margin, the buccolingual width should not be more than 1 mm wider than the cementoenamel junction,^7^ the proximal surface should be curved occlusogingivally below the contact area,^79^ and the crown margin should be merged with the tooth surface without over- or underextension and marginal gap.^38^ When a furcation area is exposed, the contours should be extended to cover the exposed root surface to eliminate the food trap formed by the roots and the cervical prominence.^7^ If the margin is overextended, the crown should be adjusted so that a probe can pass from the tooth to the crown without a catch. However, if the margin is deficient, the prosthesis should be redone.^79^

Food impaction resulting from the failure of the prosthesis might be prevented if extra precautions are taken while designing it.^79^ For better restoration outcomes, information related to the desired contour and contact must be properly communicated to the dental technician. Conversely, when preparing the tooth for the prosthesis, clinicians should follow the original contour. If the axial surface must be flat, the dental technician may need to make an overcontoured crown, if there was no other choice.^24^

Radafshar et al^96^ conducted a long-term study on participants with vertical food impaction; because of defective restorations, they were treated by replacing the restorations to achieve stable proximal contacts. However, in 10 years, approximately 25% of the treated restorations still failed to prevent vertical food impaction at the interproximal contact, and the stability of the re-treated faulty restorations in patients with vertical food impaction was 66%–89%. In addition, cusp/marginal ridge occlusal contact and patients’ age were indicated as predictive factors for the failure of proximal contact maintenance. In patients aged >40 years, the interproximal contact tightness may be lower because of tooth movement during function, caused by occlusal wear or age-related changes in bone density and height. Thus, special attention must be paid to the occlusal pattern and patients’ age while periodically evaluating the restorations in terms of proximal surfaces.^96^ The occlusal force and craniofacial growth may play a role in this occurrence. Effective treatment of vertical food impaction has remained a challenge. Future studies are needed to address the risk factors of proximal contact instability.

#### Positional Abnormality

Rotated, tilted, and partially impacted teeth will alter the proximal relationship with adjacent teeth and incline the occlusal surface. Malaligned teeth may need orthodontic treatment. Tooth crowding and rotations are regularly corrected by orthodontic treatment using initial arch wires with fixed appliances.^115,116^ In addition, after tooth extraction, prosthetic rehabilitation should be conducted promptly to prevent the adjacent teeth from drifting. In the case of drifting teeth, sufficient space must be created for the prosthesis, and the tilted teeth must be straightened by orthodontic treatment.^30,70^ Furthermore, to manage impacted third molars, orthodontic repositioning, surgical straightening, and extraction can be performed depending on the severity of the impaction.^104^

#### Interdental Papilla Loss

In dentistry, the regeneration of interdental papilla loss is one of the least predictable and most challenging treatments.^126^ Abnormal tooth form, faulty restoration contours, and excessive oral hygiene may negatively influence the shape of the interdental papillae.^95^

Various methods can be used to manage papilla loss, such as surgical procedures, nonsurgical procedures (restorative and orthodontic treatments), and minimally invasive procedures (laser and injection). Surgical techniques should aim to regenerate the gingival tissue by soft tissue augmentation; meanwhile, nonsurgical techniques intend to adjust the embrasure space so that the papillae can be filled by the gingival tissue.

Depending on the amount of tissue lost, nonsurgical or surgical approaches can be performed to restore these areas. Surgical treatments are more appropriate in the presence of a large area of soft tissue recession or bone defect.^34^ Nevertheless, the outcome of this treatment is difficult to predict, depending as it does on the amount of remaining papilla and blood supply.^6,43,95^ In contrast, nonsurgical and minimally invasive treatments were believed to be more predictable and more time-effective; however, these procedures cannot treat a large volume of periodontal recession.^126^ Thus, there is not one method that can be applied to all cases and satisfy all requirements.

Surgical reconstruction, a sensitive technique, requires well-designed and accurately performed incisions and flaps to ensure the least possible disruption of the blood supply.^43^ The subepithelial connective tissue graft is considered the gold-standard for periodontal plastic surgery, especially in the long term.^127^ Although the use of a subepithelial connective tissue graft to reconstruct papillary recession may yield the expected results, a second surgical site is still a must.^2^ Moreover, after the operation, the donor site often makes the patients more uncomfortable than the graft site, and the volume of the donor tissues is not always sufficient to meet the requirements.^123^ Thus, in some studies, platelet-rich fibrin (PRF) was applied, with the advantages of ease of procurement, lower cost, better healing for the surgical site, and no need for a second surgical site.^73^ PRF application achieved stability as well as predictable and successful outcomes in the management of papilla recession.^2^ In addition, some synthetic materials have been used. One study reported long-term assessment of grafting a synthetic biomaterial, finding stable interproximal soft tissues and patient satisfaction.^16^

Regarding nonsurgical approaches, orthodontic therapy and restorative treatment can be applied to improve the interdental black triangle. However, orthodontic treatment has the potential to result in deficient interdental papillae, especially in cases where teeth overlap. This dilemma might be due to inappropriate bracket position, alveolar bone absorption, or increased tooth crown length.^126^ While conducting restoration to reduce the interdental black triangle area, a square-shaped crown associated with the thick gingival biotype, flat, broad proximal surfaces, and contact points located as gingivally as possible have been reported to be more favourable for filling in the embrasure space.^76,91^ However, the morphological design of the prosthesis must be monitored, since changing the position of the proximal contact point and the morphological characteristics might lead to plaque accumulation and periodontal lesions.^121^

Studies have reported that local injection of hyaluronic acid (HA) adds to the benefits of nonsurgical and surgical periodontal treatment, owing to its anti-inflammatory, antibacterial, and anti-oedematous effects.^4,33,62,105^ It has been indicated as a minimally invasive, safe interproximal papilla reconstruction method that could effectively accelerate the migration and proliferation of gingival fibroblasts, reduce the size of black triangles, and increase the gingival papilla height.^57,82,83^ However, HA injections require repetition. As an alternative, the transplantation of autologous cells that can produce an extracellular matrix has been studied. Some studies have reported the potential effectiveness of tissue-engineering technology with bone marrow-derived mesenchymal stem cells on papilla augmentation in the long term. The result revealed that this technology could improve the soft-tissue aesthetics and may provide reliable outcomes.^86,123^ In addition, laser has been used widely for periodontal tissue regeneration, particularly the use of light-emitting diodes or low-level lasers on biological tissues to modulate cell function.^77^ Photobiomodulation therapy has also been used for gingival papilla regeneration.^124^ After using photobiomodulation to stimulate cell proliferation, black spaces shrank (were filled), providing improved aesthetics. Another study evaluated the effectiveness of the combination of liquid-phase concentrated growth-factor injection and low-level laser therapy.^19^ However, this combination did not show better outcomes than with either therapy alone. Further studies are needed to assess the efficacy of this combination. Drug-induced gingival hyperplasia and the use of scaffolds was also another recommendation for the treatment of horizontal food impaction.^31^ However, it was just a hypothesis, and future investigation is needed.

## Discussion

**Fig 5:**
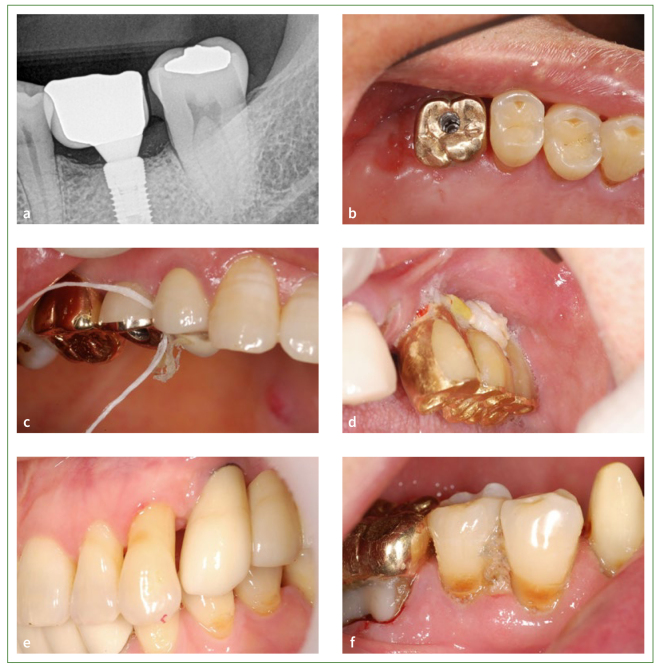
Variety of food impaction in clinical situations. (a) and (b) Proximal contact loss between implant-supported fixed dental prostheses and adjacent teeth. (c) Tight contact makes interdental cleaning more difficult. (d) Food impaction or stagnation around fixed implant supported bridge prosthesis due to the undercut made by bone resorption. (e) and (f) Interdental papilla loss favouring food impaction.

Food impaction in dentistry is stated to be one of the most common causes of carious and periodontal diseases. However, up to now, no standard method could detect or confirm the existence and severity of food impaction. Attention should be paid to identifying the presence of potential aetiological factors of food impaction (Table 1), such as PCL, occlusal disharmony, morphological deformity, positional abnormality, and interdental papilla loss. More than one factor may exist simultaneously. Furthermore, although food impaction is the common cause of periodontal disease and caries, initial decay and periodontal lesions can provoke food impaction. It is unclear whether food impaction leads to caries and periodontal disease or the other way around; however, it is more likely to happen in both ways. Either way, the complications of food impaction must be treated. In addition, if actual food impaction is not found, the success of treatment cannot be achieved. After identifying the presence of food impaction and its aetiologic factors, a specific treatment plan should be made, based on the individual patient and the complexity/variety of food impaction in clinical situations (Fig 5). The most crucial principle of food impaction treatment is to re-establish to the greatest possible extent the harmony of occlusion, shape, position of teeth, and periodontal papillae. In addition, oral hygiene plays an important role in the treatment and prevention of food impaction. Dental-hygiene education must be provided to patients with risk factors of food impaction, and interdental cleaning must receive more attention. However, appropriate force while using interdental cleaning aids should be applied, as excessive force can cause traumatic lesions and affect the interdental area.

**Table 1:** Aetiology of food impaction

Aetiology	Risk factors
Proximal contact loss	Proximal wear, tooth migration
Occlusal disharmony	Occlusal wear, plunger cusps, lack of escape grooves, uneven marginal ridges, extrusion beyond the occlusal plane, occlusal interferences, unstable contact
Morphological deformity	Tooth defect, faulty restoration
Positional abnormality	Rotated teeth, teeth tilting, partially impacted teeth
Interdental papillae loss	Alveolar atrophy, periodontal disease, trauma, aging

If potential factors are detected with neither signs nor complications of food impaction, no treatment is needed yet; however, the patients should be monitored and examined carefully, to enable early intevention when necessary. Nonetheless, in some cases of malocclusion and congenital oral anomalies, such as supernumerary teeth, mesiodens, and deep overbite, which can have severe complications, early diagnosis and treatment are recommended to avoid more serious consequences and a more complicated treatment plan. The treatment of these abnormalities can be complex and involve different disciplines, e.g. orthodontic, restorative, and surgical procedures.^9,75,108^

Nevertheless, in long-term studies, replacing restorations to achieve a stable proximal contact failed to eliminate vertical food impaction over time, and the recurrence time was shorter after each repair.^67,96^ The complexity of the masticatory system, occlusal relationships, and continuous craniofacial growth may make it difficult to manage and eliminate the vertical food impaction. To achieve a more stable outcome in the treatment and management of vertical food impaction, clinicians should take a comprehensive approach to each case.^96^ The aetiology and other related factors must be carefully examined regularly, because the interaction of these factors may lead to the occurrence or reoccurrence of vertical food impaction. The management of vertical and horizontal food impaction is complicated by various aaetiological factors and the unpredictability of treatment outcomes.

Several studies have reported that three-dimensional superimposition was an effective clinical technique to evaluate tooth movements. In addition, deep learning has been used to assess features of food impaction. These methods may present promising research directions for clinical therapy.^20,47^

## Conclusion

**Fig 6:**
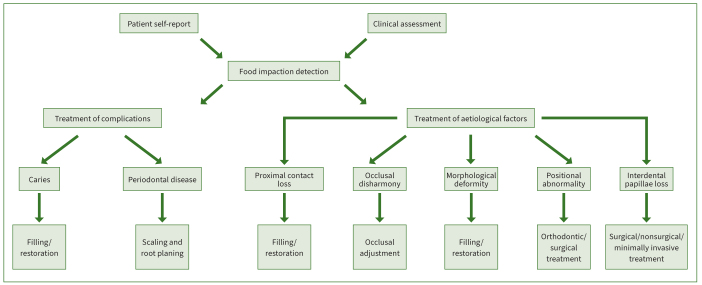
Treatment of food impaction.

This review focused on presenting the causes of food impaction along with the treatment reported through the literature (Fig 6). Various risk factors have been shown to contribute to food impaction. Owing to the complexity of food impaction, clinicians should note the reasons and related elements before treatment to optimise the effectiveness. Further studies are needed to address the prevention and management of food impaction.
